# QTL mapping and candidate gene analysis of cadmium accumulation in polished rice by genome-wide association study

**DOI:** 10.1038/s41598-020-68742-4

**Published:** 2020-07-16

**Authors:** Xiaowu Pan, Yongchao Li, Wenqiang Liu, Sanxiong Liu, Jun Min, Haibo Xiong, Zheng Dong, Yonghong Duan, Yaying Yu, Xiaoxiang Li

**Affiliations:** 1 Hunan Rice Research Institute, Hunan Academy of Agricultural Sciences, Changsha, 410125 China; 2 Key Laboratory of Indica Rice Genetics and Breeding in the Middle and Lower Reaches of Yangtze River Valley, Ministry of Agriculture, Changsha, 410125 China

**Keywords:** Agricultural genetics, Genetic association study, Plant breeding, Quantitative trait

## Abstract

Cadmium (Cd) accumulation in rice is a serious threat to food safety and human health. Breeding rice varieties with low Cd accumulation is one of the most effective approaches to reducing health risks from Cd-polluted rice. However, the genetic basis of Cd accumulation in grains, especially in *indica* rice varieties, has not been fully elucidated. The evaluation of Cd-accumulation capacity was conducted among 338 diverse rice accessions grown in Cd-contaminated soils with different Cd contents. Thirteen rice lines with relatively low Cd accumulation, including six *indica* rice lines, were identified. Then, 35 QTLs significantly associated with Cd accumulation were identified through sequencing-based SNP discovery and a genome-wide association study (GWAS) in the two experimental years, and only *qCd8-1* was detected in both years. Among of them, nine QTLs were co-localized with identified genes or QTLs. A novel QTL, *qCd1-3*, with the lowest *P * value was selected for further LD decay analysis and candidate gene prediction. We found differential expression of *OsABCB24* between high-Cd-accumulative and low-Cd-accumulative accessions, suggesting it may be a candidate gene for *qCd1-3* associated with low Cd accumulation. These results may be helpful for further exploiting novel functional genes related to Cd accumulation and developing rice variety with low Cd accumulation through marker-assisted breeding.

## Introduction

Rice (*Oryza sativa* L.) is one of the world’s staple food crops. Due to rapid industrialization and urbanization in recent years, heavy metal contamination in arable soils has become an increasingly severe problem in China. Among the heavy metals, cadmium (Cd) is the most serious contaminant. Reportedly, average Cd concentrations in paddy fields of China reached 0.23 mg/kg, and the highest concentration of 0.73 mg/kg was recorded from Hunan province^1^. Recently, Cd-polluted rice from Hunan, the largest rice-producing province in China, has become a particular concern in food safety. It has been reported that rice tends to accumulate more Cd than other cereals^2^. Moreover, the acidification of paddy soil caused by frequent applications of nitrogen fertilizer increases Cd solubility and results in more Cd absorption by rice plants^3^. In rice, Cd inhibits growth and development by causing misfolding of functional proteins and interfering the homeostasis of other essential metal ions^4^. For people, excessive accumulation of Cd may result in diseases such as cancer, anemia, heart failure, hypertension, as well as other chronic disorders^5,6^. A range of measures have been adopted to reduce Cd bioaccumulation, including soil remediation, Cd immobilization and transgenic techniques. The most promising strategy is to screen and breed rice varieties with low Cd accumulation, especially crops grown on slightly to moderately Cd-contaminated soils^2^.

Genetic variation naturally occurs among rice varieties in their abilities to accumulate Cd. Thus, extensive screening has been carried out to identify rice genetic resources with low Cd accumulation^7–9^. In general, *indica* rice varieties accumulate higher amounts of Cd in the grains than do *japonica* rice varieties^9,10^. *Indica* rice varieties are grown predominantly in southern China, and their planting areas coincide with the high-Cd-contaminated region, which further increases the risk of greater Cd accumulation in rice. Using populations derived from crosses between *indica* and *japonica* varieties, many quantitative trait loci (QTLs) for Cd accumulation have been identified^11–15^. Ueno et al.^7^ detected a major QTL (*qCd11*) controlling the Cd concentration in shoots using a F_2_ population. Liu et al.^16^ identified seven QTLs and validated *qCd2* associated with grain Cd content using a recombinant inbred line (RIL) population. Another major QTL (*qCd7*) was repeatedly detected using different genetic populations and finally cloned^17–20^. The candidate gene of *qCd7*, *OsHMA3*, encodes a tonoplast-localized P_1B_-type ATPase. OsHMA3 is involved in Cd sequestration from the cytoplasm into the vacuoles of root cells and its dysfunction promotes root-to-shoot Cd translocation and consequently increases Cd accumulation in the shoots and grains in some varieties^18,19^. Several dysfunctional alleles of *OsHMA3* have been identified in different *japonica* rice accessions^21,22^. However, one SNP in the promoter of *OsHMA3*, which alters the normal expression of *OsHMA3*, is responsible for the differential Cd accumulation between *indica* and *japonica* rice varieties^23^. Like the function of OsHMA3, ATP-binding cassette (ABC) proteins mediate Cd sequestration and confer Cd tolerance in *Arabidopsis thaliana*^24,25^.

Most of the Cd-related QTLs were identified using bi-parental populations. However, the progress of QTL mapping is hindered due to limited allele diversity and less recombination in bi-parental populations^26^. Genome-wide association study (GWAS) can overcome these two limitations and is a powerful tool to identify genome regions associating with complex traits^27,28^. Through a GWAS of 276 diverse accessions, 60 QTLs were detected for accumulation of arsenic, cadmium, and lead in rice^29^. Zhao et al.^30^ identified 14 QTLs associated with Cd accumulation in rice by GWAS and predicted *OsNRAMP2* as the candidate gene of *qCd3–2*. Using a composite method combining GWAS and other analyses, Yan et al.^10^ found that a missense mutation in *OsCd1* resulted in the *indica*-*japonica* differentiations of Cd accumulation in rice grain. These reports clearly demonstrate that GWAS is an effective approach to elucidate the genetic mechanism underlying Cd accumulation. Unfortunately, for HMA3 and most of these Cd-related QTLs, the favorable alleles for reducing Cd accumulation were basically derived from *japonica* rice varieties and therefore limited their breeding application in *indica* rice.

To identify rice germplasms with low Cd accumulation and the responsible loci for Cd accumulation, we selected 338 accessions mainly composed of *indica* rice to evaluate their Cd accumulation in different Cd-polluted paddy fields. 13 rice lines, including six *indica* rice lines, were identified as low Cd-accumulative germplasms. Based on the specific-locus amplified fragment sequencing (SLAF-seq) method^31^, genome-wide SNP discovery and a GWAS strategy were used to identify QTLs associated with Cd concentration in polished rice. 35 QTLs significantly associated with Cd accumulation were identified in two experimental years, but only *qCd8-1* was detected in both years. Through a combined analysis of LD decay and gene expression, we predicted *OsABCB24* as the candidate gene of *qCd1-3*. These results will be helpful to elucidate the genetic mechanism of Cd accumulation and provide a good basis for breeding low Cd-accumulative *indica* rice varieties.

## Results

### SLAF-based SNPs discovery among rice accessions

After sequencing and quality control, a total of 688,782 SLAF tags were obtained for each of the 338 accessions, and 515,447 polymorphic SLAFs were identified by conducting sequence alignment with the *93-11* reference genome (Supplementary Table 1). The average sequence depth was 14.3× , ranging from 8.7 to 25.5 × among different accessions. Using the GATK and Samtools software packages, 3,960,919 SNPs were called from the SLAFs for the 338 genotypes. Based on the criterion of having MAF larger than 0.05 and missing genotype rate less than 0.2, only 123,865 SNPs (3.11%) passed filters from the SNP dataset and were used for subsequent analysis. These high-quality SNPs were evenly distributed on 12 chromosomes with average density of approximately one SNP per 3.02 Kb (Supplementary Table 2). The highest maker density was detected on chromosome 7 (one SNP per 2.63 Kb), while the lowest density was detected on chromosome 8 (one SNP per 3.55 Kb).

### Population structure and relative kinship

**Figure 1:**
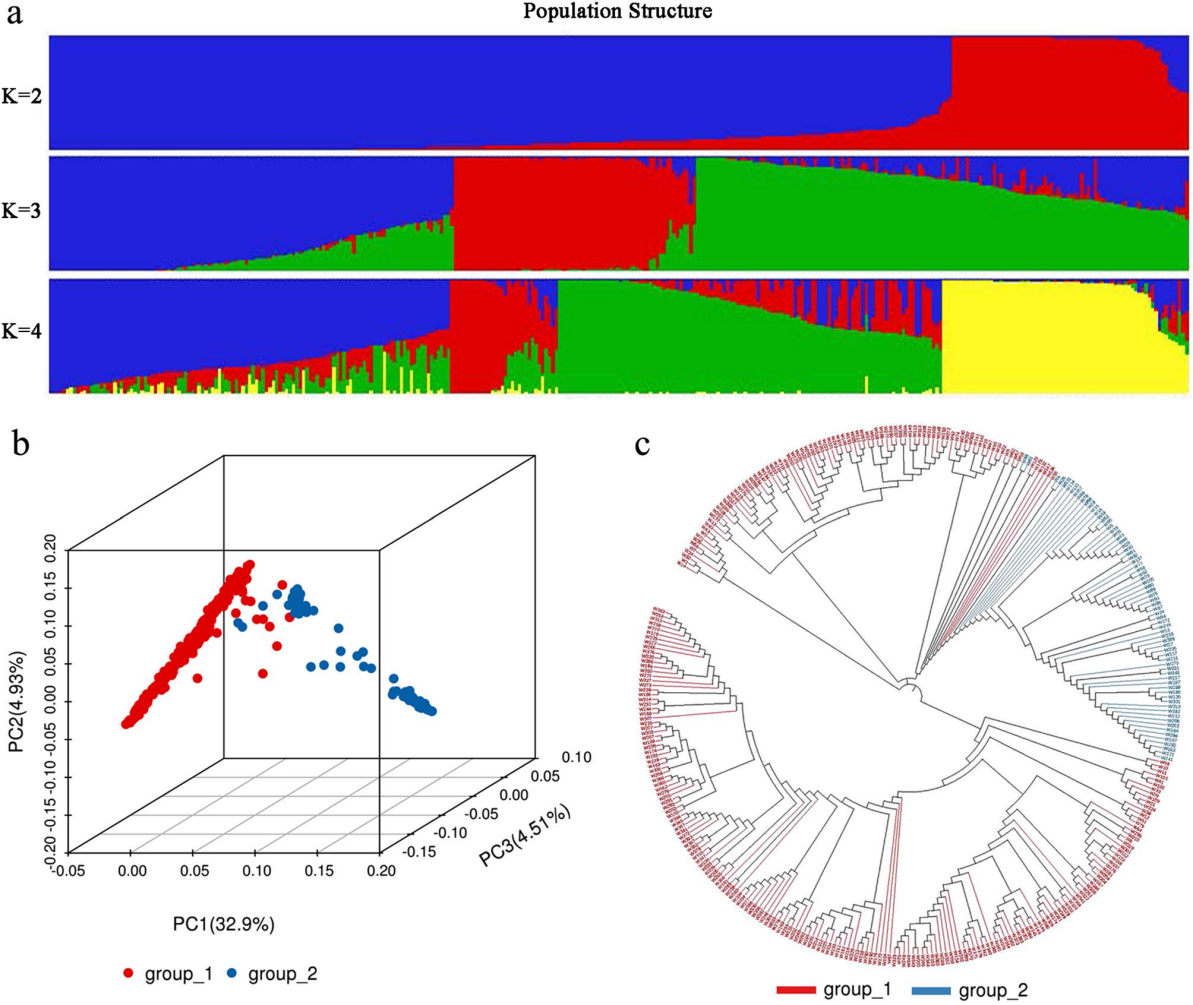
Analysis of population structure (**a**), principal component (**b**) and phylogenetic tree (**c**) for 338 rice accessions. Group_1 in red indicates the *Indica* subgroup, while group_2 in blue indicates the *Japonica* subgroup.

Population structure analysis can provide information on the origin and composition of individuals. Based on the filtered high-quality SNPs, the optimal number of ancestors (K) was estimated using the STRUCTURE software. The ∆K value was lowest when K was set to 2, suggesting that the whole group of rice lines could be divided into two subgroups (Fig. 1a). Consistently, the Principal Component Analysis (PCA) results showed that two clusters clearly separated along the eigenvector of PC1, which accounted for 32.9% of the genetic variation (Fig. 1b). Based on the neighbor-joining algorithm, a phylogenetic tree for each sample was constructed with two subgroups (1 and 2) (Fig. 1c). Subgroup_1 contained 268 rice lines, which were in accordance with the *indica* subspecies. Subgroup_2 contained 70 lines, including a portion of the foreign germplasm and landraces, which were in accordance with the *japonica* subspecies. Most of the breeding accessions and landraces belonged to subgroup_1. Interestingly, glutinous lines of landraces genetically diverged significantly, with 15 and 20 lines classified to subgroup_1 and subgroup_2, respectively. Moreover, there was differentiation within the *indica* rice subgroup. Most of the bred varieties had close relationships with foreign *indica* germplasms but were distantly related to the landraces, indicating that these landraces were rarely used in modern rice breeding in China.

### Variation of Cd accumulation in polished rice among 323 lines

**Figure 2:**
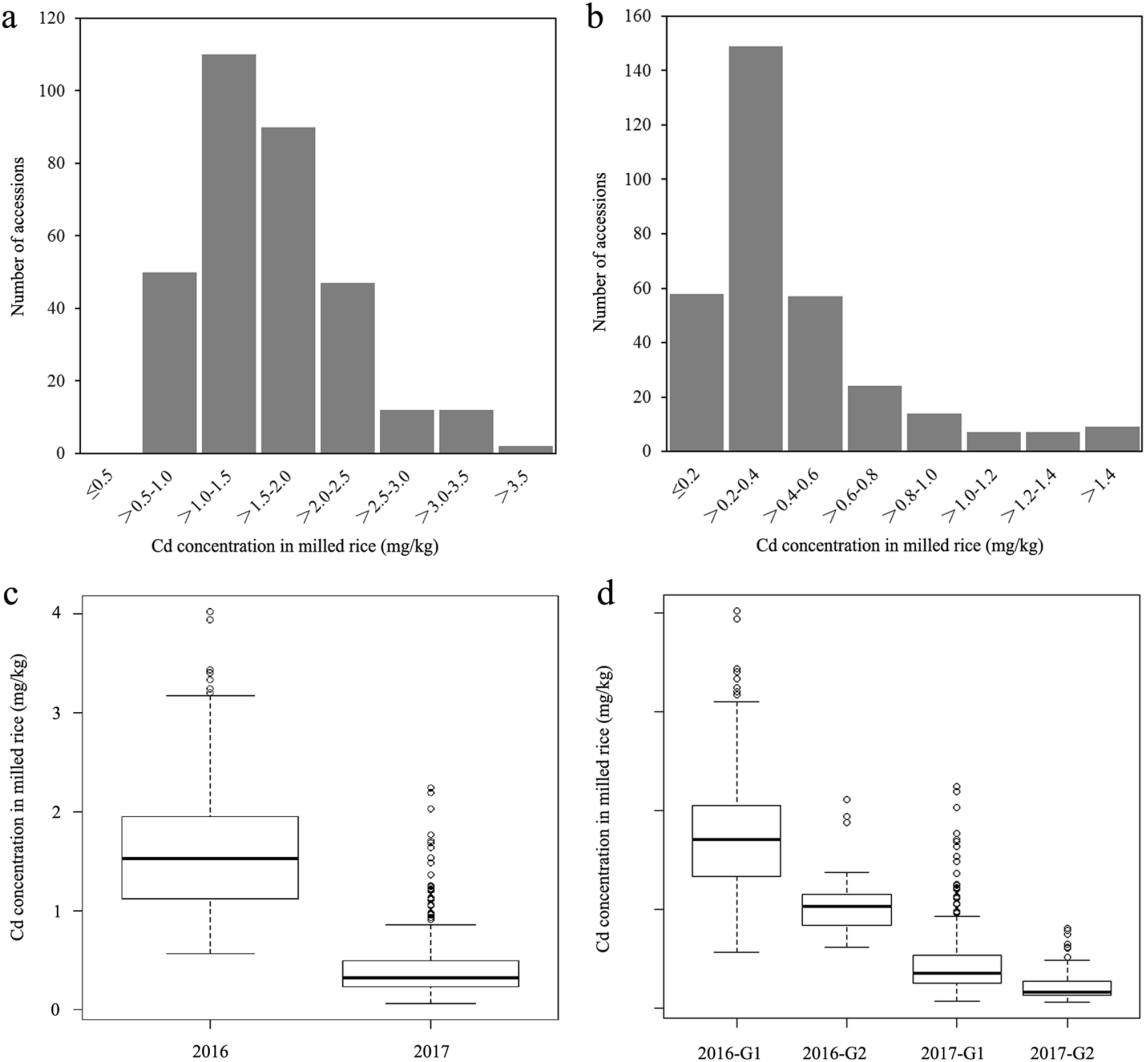
Distribution of Cd concentration in polished rice. (**a**) Cd concentration of polished rice in 2016; (**b**) Cd concentration of polished rice in 2017; (**c**) Boxplot of Cd accumulation in 2016 and 2017; (**d**) Boxplot of Cd accumulation in different subgroups in 2016 and 2017, G1 and G2 indicate group_1 and group_2 respectively.

In order to reduce confounding effects from variable growth durations among accessions, only 323 lines with moderate durations of growth were selected for Cd determination and GWAS analysis. The Cd concentrations of polished rice collected from Cd-polluted fields were determined using atomic-absorption spectrometry. The Cd accumulation varied significantly among different rice accessions in both years (Supplementary Table 3). In 2016, Cd concentrations in polished rice ranged from 0.57 mg/kg to 4.03 mg/kg, with an average of 1.61 mg/kg and a median of 1.53 mg/kg (Fig. 2a). All the rice lines showed higher Cd accumulation over 0.2 mg/kg, which is the allowable concentration for human consumption as stipulated by the National Food Hygiene Standard of China. In 2017, Cd concentrations ranged from 0.06 mg/kg to 2.24 mg/kg, with an average of 0.43 mg/kg and a median of 0.33 mg/kg (Fig. 2b). Only 58 lines displayed Cd concentrations less than 0.2 mg/kg, accounting for 18.0% of all rice lines. Overall, rice Cd accumulation in 2017 was significantly lower than that in 2016, indicating that Cd concentrations of soil was a key factor determining Cd accumulation in grains (Fig. 2c). Despite large differences between two years, thirteen rice lines were identified with relatively low Cd accumulation (Cd2016 < 0.8 mg/kg and Cd2017 < 0.2 mg/kg) in both years (Table 1). For example, breeding material BS114 showed the lowest Cd accumulation (0.57 mg/kg) in 2016 and also extremely low Cd (0.07 mg/kg) in 2017, respectively. Additionally, many landraces from Hunan province, such as “Shenshuinuo” and “Daganzaogu,” were found in this low-Cd-accumulative group and could be used as potential donors in future low-Cd rice breeding.

**Table 1:** List of some low-Cd-accumulative rice accessions.

Field ID	Cd-2016	Cd-2017	Accession name	Type	Origin
W132	0.57	0.07	BS114	Breeding material	China
W275	0.62	0.14	Shenshuinuo	Landrace	China
W393	0.63	0.12	Daganzaogu	Landrace	China
W31	0.66	0.06	IAC25	Foreign germplasm	Brazil
W394	0.68	0.18	Hongjiaozao	Landrace	China
W135	0.69	0.09	BS82	Breeding material	China
W172	0.69	0.09	Changgu	Landrace	China
W216	0.70	0.12	Hongsanlicun	Landrace	China
W86	0.73	0.13	U4	Foreign germplasm	Uruguay
W377	0.73	0.11	Zaomagu	Landrace	China
W392	0.73	0.17	Caohezi	Landrace	China
W367	0.78	0.17	Xiyesu	Landrace	China
W217	0.79	0.13	Hongtaonuo	Landrace	China

Considering the presence of distinct population structure, Cd accumulations were compared between different subgroups in both years. As shown in Fig. 2d, Cd accumulation in the *indica* subgroup was significantly higher than that in the *japonica* subgroup (*P* < 0.001). In 2016 and 2017, the mean Cd accumulations in the *indica* subgroup were 1.75 mg/kg and 0.47 mg/kg respectively, while in the *japonica* subgroup were 1.04 mg/kg and 0.25 mg/kg, respectively. These results clearly indicated that population structure had effect on the Cd accumulation in these rice lines.

### GWAS for Cd accumulation

**Figure 3:**
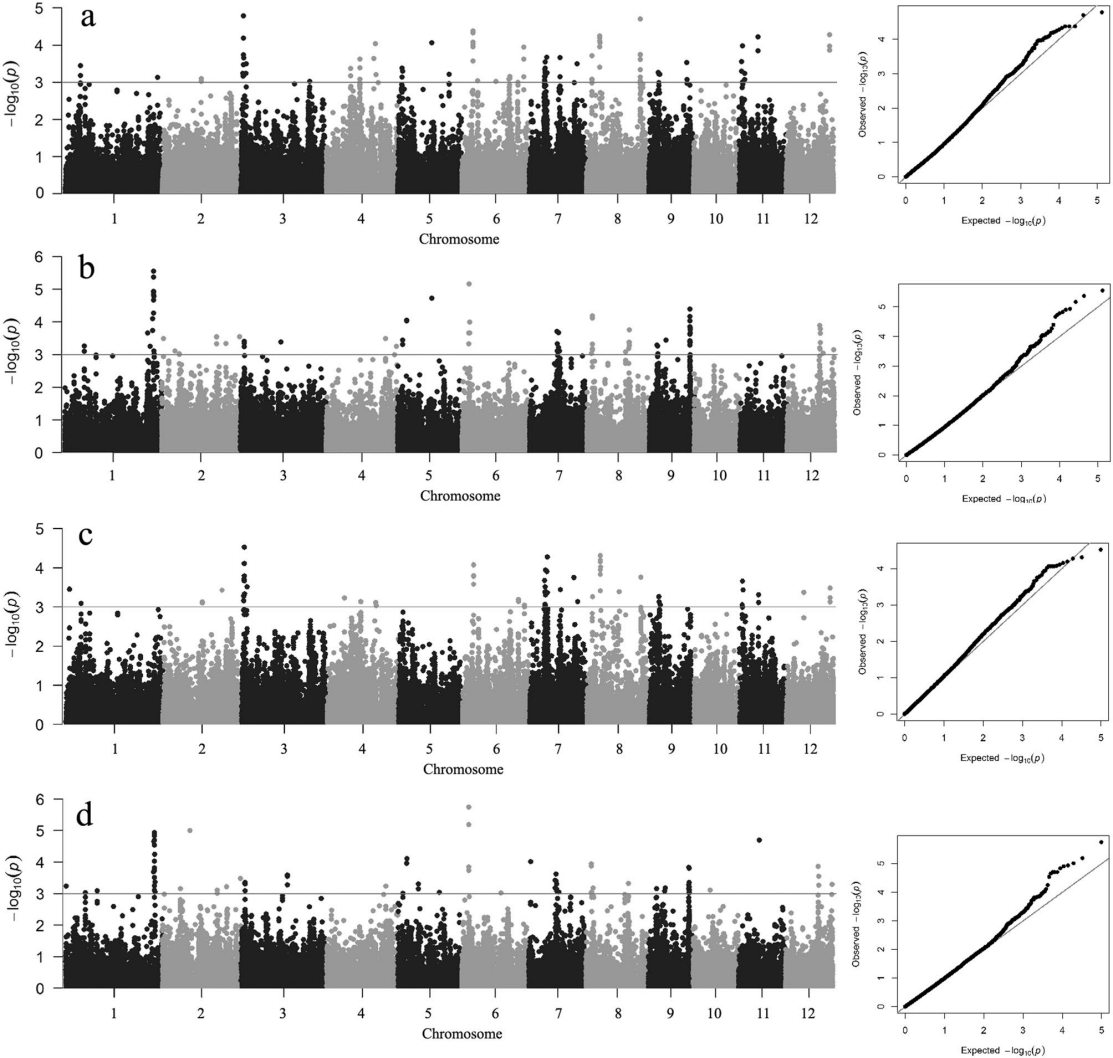
Manhattan plots and quantile–quantile (Q–Q) plot of GWAS for Cd accumulation. (**a**) Manhattan plots and Q-Q plot of GWAS for Cd accumulation in 2016 using the whole group; (**b**) Manhattan plots and Q-Q plot of GWAS for Cd accumulation in 2017 using the whole group; (**c**) Manhattan plots and Q-Q plot of GWAS for Cd accumulation in 2016 using the *Indica* subgroup; (**d**) Manhattan plots and Q–Q plot of GWAS for Cd accumulation in 2017 using the *Indica* subgroup. The horizontal line in Manhattan plots indicates threshold of *P* value.

To investigate the genotypic basis underlying Cd accumulation in polished rice, we performed GWAS to identify the associated SNP loci in the selected 323 rice lines. Considering the effect of population structure on Cd accumulation, the mixed linear model (MLM) model was adopted with kinship matrix and PC matrix as covariates. According to Lv et al.^32^, a region was considered as one QTL when more than two significant SNPs (*P* < 0.001) were detected within a 200-Kb window. In total, 35 QTLs with 203 SNPs significantly associated with Cd accumulation were identified in the two experimental years with a well-fitted quantile–quantile (Q–Q) plots (Table 2, Supplementary Table 4, Fig. 3a,b). These QTLs were distributed on all chromosomes except chromosome 10. The comparison of QTLs identified in two years indicated that only *qCd8-1* was detected in both years, suggesting that environmental factors might have great influence on the GWAS results of Cd accumulation. To verify the accuracy of the GWAS results, the identified QTLs in this study were further compared with previous reports. We found 9 QTLs were co-localized with previous mapped QTLs, associated markers and characterized genes (Table 2), indicating that GWAS results were reliable in this study. Among the co-localized QTLs, *qCd6-2* on chromosome 6 was located close to *OsLCT1*^33^, and *qCd7-1* on chromosome 7 was identified in the genome interval of the well-characterized gene *HMA3*, which is involved in Cd transport into rice grains. The remaining QTLs have not been reported previously and were considered as novel QTLs.

**Table 2:** The mapped QTLs for Cd accumulation in polished rice.

QTLs	Chr	Position of lead SNP	*P* value	Known loci
**2016**				
*qCd1-1*	1	7426674	3.59E−04	
*qCd2-1*	2	19009363	7.91E−04	
*qCd3-1*	3	1379311	1.62E−05	RM132, Zhang et al.^15^; Sun et al.^9^
*qCd4-1*	4	11703239	4.23E−04	
*qCd4-2*	4	16185795	2.38E−04	*qCCBR4-3*, Hu et al.^12^
*qCd5-1*	5	2071616	4.15E−04	
*qCd6-1*	6	5455316	4.15E−05	
*qCd6-2*	6	23453239	6.91E−04	*OsLCT1*, Uraguchi et al.^33^
*qCd6-3*	6	30153194	1.13E−04	
*qCd7-1*	7	7701584	2.82E−04	*HMA3*, Ueno et al.^18^; RM8006, Sun et al.^9^
*qCd7-2*	7	14958252	2.18E−04	
*qCd8-1*	8	2603062	1.94E−04	
*qCd8-2*	8	6474266	5.64E−05	
*qCd8-3*	8	26140129	1.97E−05	RM149, Zhang et al.^15^
*qCd9-1*	9	4561580	5.42E−04	
*qCd9-2*	9	18482205	2.95E−04	
*qCd11-1*	11	1326077	2.78E−04	
*qCd11-2*	11	1670098	1.04E−04	
*qCd11-3*	11	2744623	5.73E−04	
*qCd11-4*	11	9253294	6.00E−05	
*qCd12-1*	12	21131708	5.21E−05	*qCCBR12-2*, Hu et al.^12^
**2017**				
*qCd1-2*	1	9595068	5.43E−04	
*qCd1-3*	1	43287290	2.78E−06	
*qCd2-2*	2	8591863	9.46E−04	
*qCd2-3*	2	26617405	2.83E−04	
*qCd3-2*	3	1994025	3.90E−04	
*qCd5-2*	5	2429263	3.62E−04	
*qCd5-3*	5	4299965	8.88E−05	
*qCd6-4*	6	3510600	6.86E−06	
*qCd7-3*	7	13490007	4.68E−04	RM7273-RM5481, Abe et al.^11^
*qCd7-4*	7	14106550	2.12E−04	RM7273-RM5481, Abe et al.^11^
*qCd8-1*	8	2585361	6.61E−05	
*qCd8-4*	8	20537115	1.77E−04	
*qCd9-3*	9	19685615	4.01E−05	RM215, Sun et al.^9^
*qCd12-2*	12	15813626	1.28E−04	

To further exclude interference from population structure, GWAS was conducted using the *indica* subgroup of 259 accessions and compared with the identified QTLs using the whole group (Fig. 3c,d, Supplementary Table 5). The *japonica* subgroup was not analyzed separately in this study due to a limited number of rice lines. For the *indica* subgroup, GWAS results were basically consistent with those for the whole group. However, 16 QTLs including *qCd1-1*, *qCd1-2*, *qCd2-2*, *qCd4-1*, *qCd4-2*, *qCd5-1*, *qCd5-2*, *qCd6*-2, *qCd7-2*, *qCd7-4*, *qCd8-1*, *qCd8-3*, *qCd8-4*, *qCd9-2*, *qCd11-2* and *qCd11-3* were not detected in this subgroup mainly because only one significant SNP was identified in most of these loci. In total, 19 QTLs for Cd accumulation were identified in both the whole group and *indica* subgroup. Among these QTLs, *qCd1-3* showed the lowest *P *value on chromosome 1 near position 43.3 Mb and was chosen for subsequent analysis.

### Identification of candidate genes responsible for Cd accumulation

**Figure 4:**
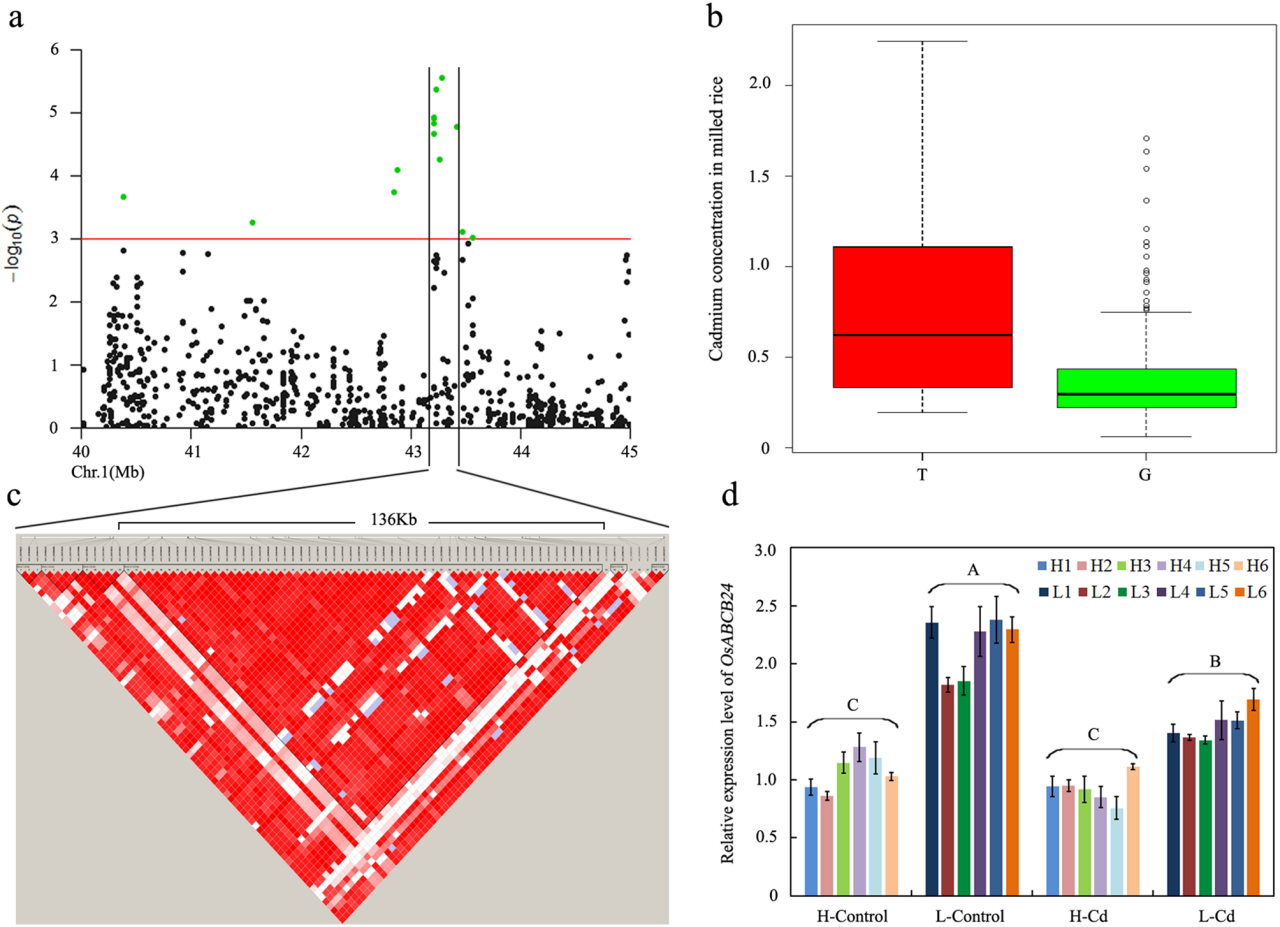
Candidate gene estimation of *qCd1-3* on chromosome 1. (**a**) Local manhattan plots of GWAS for Cd accumulation around *qCd1-3*; (**b**) Distribution of Cd concentration in polished rice between the favorable allele G and the unfavorable allele T; (**c**) LD heatmap of around *qCd1-3*; (**d**) Expression of *OsABCB24* between high-Cd-accumulative and low-Cd-accumulative rice accessions. Varieties accessions, H1: W261; H2: W233, H3: W32; H4: W357; H5: W333; H6: W278; L1: W49; L2: W30; L3: W132; L4: W18; L5: W135; L6: W95. Different letters indicate significant difference at 1% level (Duncan’s multiple range test).

Around the interval of *qCd1-3*, 14 consecutive SNPs were significantly associated with Cd accumulation in 2017 (Fig. 4a), among which the lead SNP *rs1_43287290* (*P* = 2.78E−06) was selected as the representative of this loci. According to the alleles of the lead SNP, all samples were divided into two groups of a favorable allele G (designated as group G) and an unfavorable allele T (designated as group T) respectively. Cadmium accumulation in group G was significantly lower than that in group T. An average of 0.78 mg/kg and a median of 0.62 mg/kg were observed in group T while an average of 0.36 mg/kg and a median of 0.30 mg/kg were observed in group G. (Fig. 4b). In order to accurately estimate the target interval, LD decay analysis was performed for the region around *qCd1-3*. With r^2^ = 0.8 as the threshold, a 136-Kb block containing the lead SNP was identified as the candidate region (Fig. 4c). Based on the annotation of the reference genome, 22 genes were identified in this block including 17 functional protein-coding genes and five lncRNA-encoding genes. One of these genes (*OsABCB24*) located approximately 71 Kb from the lead SNP was annotated as an ATP-binding cassette (ABC) transporter. Since the ABC transporter had been reported to mediate vacuolar compartmentation of Cd in root tissue, *OsABCB24* was regarded as the primary candidate gene of *qCd1-3* associated with Cd accumulation. Then we analyzed the expression of *OsABCB24* in different tissues at the vegetative stage. Although *OsABCB24* showed the highest expression in leaf, its expression in root was higher than those in many other tissues such as stem, leaf sheath and panicle (Supplementary Fig. 1). Based on the genotype of *qCd1-3*, twelve accessions with contrasting Cd accumulation, including six high-Cd-accumulative accessions and six low-Cd-accumulative accessions, were selected for further expression analysis. As shown in Fig. 4d, *OsABCB24* showed lower transcript levels in the high-Cd-accumulative rice accessions than those in the low-Cd-accumulative accessions under normal growth conditions. A similar trend was also observed under Cd-treatment conditions, even though Cd treatment slightly reduced the expression of *OsABCB24* in low-Cd-accumulative accessions. These results suggest that *OsABCB24* might be a good candidate gene for *qCd1-3*.

## Discussion

Natural genetic variation is a powerful resource not only for rice breeding but also for investigating the genetic mechanism of complex traits. Cadmium accumulation varied considerably among different rice accessions, suggesting that it is feasible to breed low Cd-accumulative rice varieties. In order to accurately evaluate the Cd-accumulation capacity among different genetic rice lines, we cultivated 338 rice lines in paddy fields that naturally contained different concentrations of cadmium, and the experiment was conducted in two consecutive years. The difference of soil Cd concentration and Cd accumulation of polished rice in 2017 was significantly lower than those in 2016, respectively. These results were consistent with the previous study that the grain Cd accumulation was largely affected by the soil Cd concentration^9^. Zhao et al.^30^ planted 312 rice accessions in a field with a pH value of 5.5 and soil Cd level of 1.4 mg/kg (similar to the Cd level in 2016 of this study), while they recorded relatively lower Cd accumulation in the grain. Because pH is probably the most important influencing factor of Cd uptake in rice plants^34^, it is reasonable to ascribe this difference in grain Cd content to the difference in pH. Consistent with previous studies^7–9^, there are wide variations in Cd accumulation levels among different rice accessions. In addition, our results also indicted that *indica* rice accessions tended to accumulate more Cd than *japonica* accessions. Nevertheless, several *indica* rice lines with relatively low Cd accumulation were identified in both years, most of which are landraces from Hunan province. After long-term evolution under natural and artificial selection, rice landraces show high genetic diversity and outstanding environmental adaptability^27^. The results showed that most of the landraces were genetically distant from the bred varieties, and they could be ideal donors for breeding low-Cd-accumulative rice varieties, especially for *indica* rice varieties. Unfortunately, most of the rice lines showed higher Cd accumulation over 0.2 mg/kg in the two-year experiment. Thus, identifying accessions with relatively low Cd accumulation might be an important step towards reducing Cd risks to rice consumers.

Identifying the loci associated with complex traits in rice is challenging due to high population differentiation^26^. In this study, the population structure of the rice accessions likely affected Cd accumulation in the polished rice. MLM model has been widely adopted due to its effectiveness in controlling confounding factors and reducing the number of false positives^35^. Results of the GWAS for the *indica* subgroup were basically consistent with those for the whole group, demonstrating that the use of relatedness matrixes as covariates in GEMMA could eliminate confounding effects of population structure. The slight difference between the subgroup and whole group might be caused by two reasons: (1) a smaller number of SNPs were used in the GWAS for the *indica* subgroup; and (2) the criterion of requiring more than two significant SNPs existing within a 200-Kb window to identify a QTL may have been too strict. Another common problem encountered in the GWAS was the large effect of environment, especially when mapping isonomic traits^36^. Among the identified QTLs, only *qCd8-1* was detected under different soil Cd concentration in both years. Except for the inaccurate phenotypic identification caused by environmental factors, it’s quite likely that the genetic mechanisms underlying Cd accumulation might be divergent under different levels of Cd-polluted soils. Since the Cd pollution level of the paddy field in 2017 is more similar to the common level occurring in China^1^, the QTLs identified in this environment is more valuable for use in rice breeding than those identified in 2016.

The transfer of Cd from soil to grain is controlled by at least four steps: transport of Cd from soil into root cells, sequestration of Cd into the vacuoles, xylem loading, and phloem-mediated Cd transport to grains^37^. Nramp5 is a major transporter responsible for the Cd uptake from soil, and its knock-out resulted in a significant reduction of Cd accumulation in different genetic backgrounds of rice^38^. However, no natural allelic variation of *OsNramp5* have been reported among different genetic resources. Through GWAS analysis, Zhao et al.^30^ predicted that another homolog *OsNRAMP2* might be involved in Cd uptake in rice. OsHMA3, which specifically sequesters Cd into the vacuole of root cells and prevents its upward transport, was identified as the candidate gene for *qCd7-1* in this study. Interestingly, *qCd7-1* was also detected in the *indica* subgroup, implying that there might be natural variations of *OsHMA3* among different *indica* rice varieties. Unlike the function of *OsHMA3*, another homolog *OsHMA2* functions in the transport of both zinc (Zn) and Cd between root and shoot tissues through xylem loading^39^. The last Cd translocation step from shoot to grain involves a low-affinity cation transporter, OsLCT1, the encoding gene of which was co-localized with *qCd6-2* (identified in the present study).

Our bioinformatics and gene expression analyses suggest that *OsABCB24* was the candidate gene of *qCd1-3*. The ABC transport family is one of the largest protein families and conserved in all organisms. There are more than 125 ABC transporters in the rice genome, and their functions have yet to be elucidated^40^. Several studies have shown that ABC transporters may play important roles in Cd tolerance in plants^24,25^. Among the genes within the block of *qCd1-3*, only *OsABCB24* was identified to encode a transporter. Reportedly, ABC transporters contribute to detoxifying cadmium by pumping it into vacuoles in yeast^41^. In *Arabidopsis*, AtABCC3 functions as a transporter of phytochelatin–Cd complexes into vacuoles^42^, similar to the function of OsHMA3 in Cd vacuolar sequestration. Our results showed that the expression of *OsABCB24* was relatively high in root, and was significantly lower in high-Cd-accumulative rice accessions than that in low Cd-accumulative accessions. Because overexpression of OsHMA3 decreased Cd concentration in shoots, we proposed that the strong expression of *OsABCB24* might contribute to enhancing vacuolar compartmentation of Cd in roots, thereby reducing Cd accumulation in rice grains of low Cd-accumulative accessions. Future work will apply functional genomics methodologies such as genetic transformation and CRISPR-cas9 technology to verify the role of *OsABCB24* in regulating Cd accumulation.

## Methods

### SLAF-seq, sequencing data analysis and SNP calling

A core collection of 338 rice accessions were selected from the genetic resources in the gene bank of Hunan province, China. The whole group was composed of 148 landraces, 92 introduced foreign germplasms, 82 bred varieties and 16 breeding intermediate materials (Supplementary Table 3). Young leaves from each of the 338 accessions were collected, frozen in liquid nitrogen, and used for DNA extraction. Genomic DNA was isolated using the cetyltrimethyl annonium bromide (CTAB) protocol^43^. The SLAF libraries were constructed for each accession following the method proposed by Sun et al.^31^, and sequencing was performed on a HiSeq 2500 system (Illumina, CA, USA). The library construction and sequencing were carried out at Biomarker Technologies Corporation (Beijing, China). Because most of the rice lines belong to the subspecies *indica*, the pair-end reads were aligned to the reference genome of *indica* rice *93-11* (https://rice.genomics.org.cn/) using the MEM algorithm of Burrows-Wheeler Aligner (BWA) software (version 0.7.10)^44^. After alignment, SNP calling was conducted by the combined use of GATK (version 3.7)^45^ and Samtools (version 1.9)^46^. The identified SNPs were further filtered by the Plink software (version 1.90)^47^. Only SNPs with minor allele frequencies (MAF) > 0.05 and missing genotype rates < 0.2 were retained for GWAS analysis.

### Field and pot experiments

Two years of field experiments were conducted in two separate Cd-polluted paddy fields in Beishan, Hunan province, China. The soil Cd concentration in 2016 was 1.25 mg/kg with a pH value of 5.2, while the Cd concentration in 2017 was 0.69 mg/kg with a pH value of 5.3. To reduce potentially unexpected effects of differential growth duration among accessions, sowing dates were staggered in May based on the days to maturity of each accession to ensure most lines heading at approximately the same time. The 25-d-old seedlings were transplanted in a randomized complete block design with two replications for each line. Each replication contained 16 rice plants grown in two rows with an in-row spacing of 16.7 cm and a between-row distance of 20 cm. Flooded condition was maintained in the field until mid-August. In order to increase bioavailable Cd concentration in the soil, rain-fed irrigation was mainly adopted during the grain filling stage, and flush irrigation was applied when necessary to avoid drought stress. Other field management, including fertilizer application and disease and pest control, was conducted according to standard rice farming practice.

### Sampling and Cd determination

Rice grain was harvested 35 days after heading and dried in an oven at 40℃ for three days. The Cd concentration was determined according to the Chinese National Standard (GB 5009.15-2014). Because polished rice is the main edible part of rice, Cd accumulation in polished rice was investigated in this study. Rice grains were polished and then ground into powder. Approximately 0.3–0.5 g samples were digested with a solution of nitric acid and perchloric acid (9:1 v/v). The Cd concentration in the digest solution was measured by atomic absorption spectrometry (Solaar S4; Thermo, USA).

### Phylogenetic tree construction, population structure, and principal component analysis

A phylogenetic tree of 338 lines was constructed by MEGA 5.0^48^ using the neighbor-joining method with 1000 bootstrap replicates. The population structure was analyzed using STRUCTURE software^49^. The following parameters were used for the analysis: K = 2 to 10, burn in 5000, MCMC repeat 50,000 and three replicates for each K. Then we calculated ΔK to determine the optimal K value. The software Clumpp^50^ and Pophelper^51^ were used to visualize the population structure. The Q matrix of population structure was analyzed by ADMIXTURE software^52^. Principal components analysis was carried out using EIGENSOFT^53^.

### Genome-wide association study

A software toolkit of GEMMA was used to perform association mapping according to Zhou and Stephens^54^. The standard linear mixed model was expressed as y = Wα + xβ + u + e, where y represents the phenotypic observation, W = (w1, … wc) is an n × c matrix of covariates, α is the vector of the corresponding coefficients including the intercept, x is an n-vector of marker genotypes, β is the effect size of marker, u and e represent random effects and errors, respectively. To minimize the effect of population structure, PCA matrix and kinship matrix were used as covariates in this study. *P *values of ≤ 0.001 were used as the threshold to identify significantly associated SNPs. The SNP with the minimum *P *value in a locus was considered as the lead SNP. The allele contributing to reduction of cadmium content was regarded as the favorable allele.

### Gene prediction and expression analysis

The LD heatmap around the peak SNP in GWAS was constructed using HaploView software^55^ and the candidate region was estimated using r^2^ > 0.8. The local Manhattan plot was produced using R package qqman^56^. The reference sequences of a candidate region were downloaded for gene annotation. Based on the annotations, genes related to transport of metal ion were selected as candidate genes.

For the expression analysis, the seedlings were grown in quarter-strength Hoagland solution in a growth chamber. Ten-days-old seedlings were then transferred to a nutrient solution with a cadmium concentration of 1.0 mg/kg, while the control group of seedlings continued to grow normally in the same nutrient solution without cadmium. After one week of treatment, roots were sampled and immediately frozen in liquid nitrogen. Total RNAs were isolated using Trizol Reagent (TransGen, Beijing, China) and were used for cDNA synthesis using RT SuperMix (Vazyme, Nanjing, China). Quantitative PCR was performed on a LightCycler 96 system (Roche, Rotkreuz, Switzerland) using SYBR qPCR Master Mix (Vazyme, Nanjing, China). Gene-specific primers for *OsABCB24* were 5′- TCTTTACGAGTGACCCTGACC-3′ and 5′- CTCCATACTACCGACCCGTT-3′. Actin was used as an internal control with primers 5′- CATTGGTGCTGAGCGTTTCC-3′ and 5′- AGAAACAAGCAGGAGGACGG-3’.

## Supplementary information

Supplementary Information.

Supplementary Table S3.

Supplementary Table S4.

Supplementary Table S5.

## Data Availability

The raw reads of 338 rice accessions generated in this study have been deposited in the Sequence Read Archive (SRA) database (https://www.ncbi.nlm.nih.gov/sra) under the accession number of PRJNA629658.
