# Supplementary material for: QTL mapping and candidate gene analysis of cadmium accumulation in polished rice by genome-wide association study
Source: Sci Rep. 2020 Jul 16;10:11791. doi: 10.1038/s41598-020-68742-4 (PMC7366680; doi:10.1038/s41598-020-68742-4)
Supplement: Supplementary file 1 — Supplementary Information. [file 41598_2020_68742_MOESM1_ESM.pdf]

QTL mapping and candidate gene analysis of cadmium accumulation in polished rice by genome-wide association study

Xiaowu Pan[a,b†], Yongchao Li [a,b†], Wenqiang Liu[a,b], Sanxiong Liu[a,b], Jun Min[a,b], Haibo Xiong[a,b], Zheng Dong[a,b], Yonghong Duan[a,b], Yaying Yu[a,b], Xiaoxiang Li[a,b*]

Includes

Supplementary Figure 1. The expression of *OsABCB24* in different organs and tissues.

Supplementary Table 1. Distribution on chromosomes of SLAF tags and polymorphic SLAF tags.

Supplementary Table 2. SNPs distribution on each chromosome.

Supplementary Table 3. Basic information and Cd accumulation of 338 rice accessions. (xlsx)

Supplementary Table 4. The significant SNPs of Cd accumulation for the whole subgroup. (xlsx)

Supplementary Table 5. The significant SNPs of Cd accumulation for the indica subgroup. (xlsx)

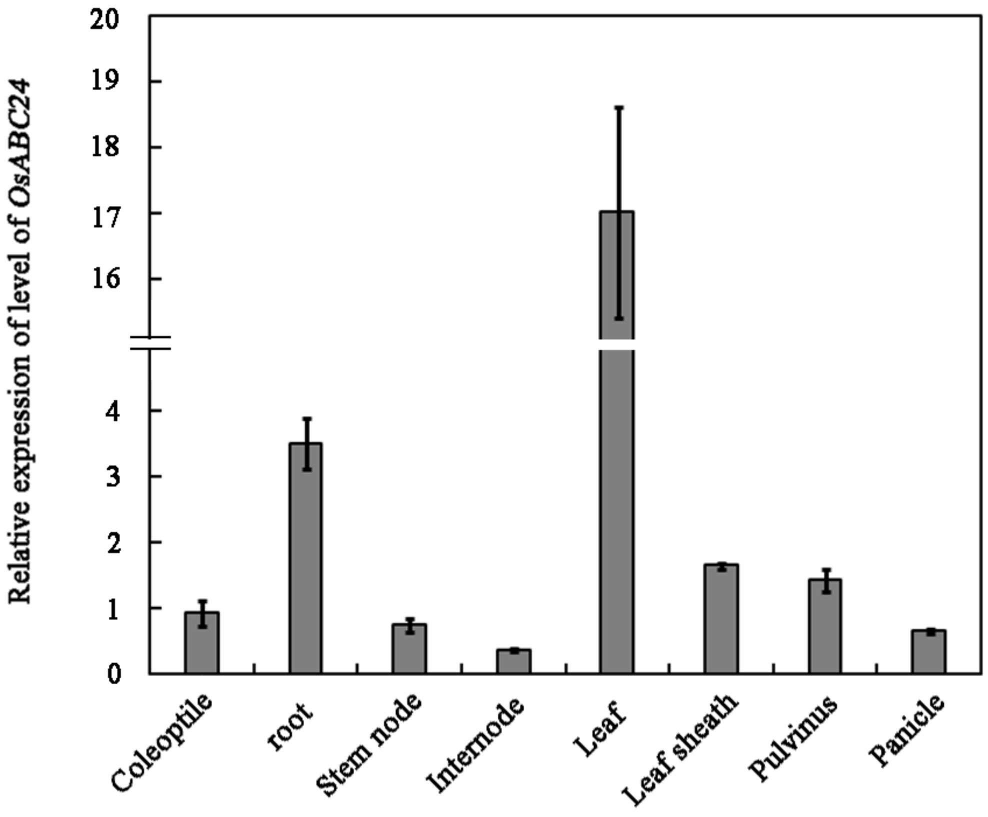

Supplementary Figure 1. The expression of *OsABCB24* in different organs and tissues.

Table S1. Distribution on chromosomes of SLAF tags and polymorphic SLAF tags.

Chromosome	SLAF tags	Polymorphic SLAF tags
1	85,732	63,739
2	72,221	54,476
3	77,429	56,639
4	63,013	45,944
5	58,406	42,979
6	59,018	44,352
7	53,002	40,750
8	54,128	39,402
9	41,448	31,872
10	40,221	30,589
11	42,234	32,853
12	41,930	31,852
Total	688,782	515,447

Table S2. SNPs distribution on each chromosome

Chromosome	SNP number	Chromosome length (bp)	SNP density(kb)
1	14962	47,244,934	3.16
2	13771	38,080,379	2.77
3	13723	41,835,883	3.05
4	10048	34,660,668	3.45
5	9806	31,162,561	3.18
6	11137	32,845,367	2.95
7	10610	27,898,288	2.63
8	8545	30,367,118	3.55
9	7719	21,712,932	2.81
10	8174	22,150,131	2.71
11	7470	22,971,669	3.08
12	7900	22,947,966	2.9
Total	123865	373,877,896	3.02
